# Peritoneal Milky Spots Serve as a Hypoxic Niche and Favor Gastric Cancer Stem/Progenitor Cell Peritoneal Dissemination Through Hypoxia-Inducible Factor 1α

**DOI:** 10.1002/stem.1816

**Published:** 2014-11-26

**Authors:** Zhi-Feng Miao, Zhen-Ning Wang, Ting-Ting Zhao, Ying-Ying Xu, Jian Gao, Feng Miao, Hui-Mian Xu

**Affiliations:** a Department of Surgical Oncology, The First Affiliated Hospital of China Medical UniversityShenyang, Liaoning Province, People's Republic of China; b Department of Breast Surgery, The First Affiliated Hospital of China Medical UniversityShenyang, Liaoning Province, People's Republic of China; c Center of Laboratory Technology and Experimental Medicine, China Medical UniversityShenyang, Liaoning Province, People's Republic of China; d Department of Digestion, The Fourth Affiliated Hospital of China Medical UniversityShenyang, Liaoning Province, People's Republic of China

**Keywords:** Stomach cancer, Peritoneal dissemination, Peritoneal milky spots, Hypoxia, Cancer stem cell, Cancer stem cell niche

## Abstract

Peritoneal dissemination is the most common cause of death in gastric cancer patients. The hypoxic microenvironment plays a major role in controlling the tumor stem cell phenotype and is associated with patients' prognosis through hypoxia-inducible factor-1α (HIF-1α), a key transcriptional factor that responds to hypoxic stimuli. During the peritoneal dissemination process, gastric cancer stem/progenitor cells (GCSPCs) are thought to enter into and maintained in peritoneal milky spots (PMSs), which have hypoxic microenvironments. However, the mechanism through which the hypoxic environment of PMSs regulated GCSPC maintenance is still poorly understood. Here, we investigated whether hypoxic PMSs were an ideal cancer stem cell niche suitable for GCSPC engraftment. We also evaluated the mechanisms through which the HIF-1α-mediated hypoxic microenvironment regulated GCSPC fate. We observed a positive correlation between HIF-1α expression and gastric cancer peritoneal dissemination (GCPD) in gastric cancer patients. Furthermore, the GCSPC population expanded in primary gastric cancer cells under hypoxic condition in vitro, and hypoxic GCSPCs showed enhanced self-renewal ability, but reduced differentiation capacity, mediated by HIF-1α. In an animal model, GCSPCs preferentially resided in the hypoxic zone of PMSs; moreover, when the hypoxic microenvironment in PMSs was destroyed, GCPD was significantly alleviated. In conclusion, our results demonstrated that PMSs served as a hypoxic niche and favored GCSPCs peritoneal dissemination through HIF-1α both in vitro and in vivo. These results provided new insights into the GCPD process and may lead to advancements in the clinical treatment of gastric cancer. Stem Cells *2014;32:3062–3074*

## Introduction

Gastric cancer is one of the most common malignant tumors in the gastrointestinal system and is associated with a pessimistic prognosis 1–3. Peritoneal dissemination frequently occurs in late-stage gastric cancer and is the most serious problem that prevents the long-term survival of gastric cancer patients 4,5. Peritoneal milky spots (PMSs) are omentum-associated lymphoid tissues that have generally been recognized as the site of origin of immature PMS macrophages 6,7. Free tumor cells in the peritoneal cavity have been shown to preferentially engraft and colonize in PMSs, resulting in peritoneal dissemination 8–10. The unique anatomical structure and vascular distribution of PMSs provide a hypoxic microenvironment that is suitable for tumor cell residence 11. It was presumed that, during gastric cancer peritoneal dissemination (GCPD), PMSs could partly eliminate mature/senescent gastric cancer cells (GCCs) by nonspecific cytotoxic effects; however, PMSs could not eliminate gastric cancer stem/progenitor cells (GCSPCs) 12,13. GCSPCs are relatively quiescence and exhibit antiapoptotic abilities in PMS hypoxic microenvironment, which could partly explain why GCSPCs can survival after traditional intraperitoneal chemotherapy and are capable of rapidly regenerating tumors, thereby leading to GCPD. Conversely, PMS macrophages can be remodeled by tumor cells, forming tumor-associated macrophages with an alternative active phenotype, which in turn support tumor cell maintenance 14.

The identification of tumor stem cell locations and regulatory mechanisms that control the maintenance of tumor stem cells is a vital prerequisite to successful antitumor strategies 15. The hypoxic microenvironment has an established role in the regulation of stem cell stemness, which can drive tumor progression by triggering a set of adaptive transcriptional responses that regulate tumor stem cell differentiation and self-renewal and ultimately promote a more aggressive tumor phenotype 16,17. These cellular responses are primarily controlled by the transcription factor system of hypoxia-inducible factors (HIFs) 18–21. HIF-1 is a heterodimeric transcription factor that consists of two subunits. The HIF-1β subunit is constitutively expressed, while the HIF-1α subunit is regulated by oxygen levels. It is stable under hypoxic conditions, but is rapidly degraded under normoxic conditions 22. After stabilization or activation, HIF-1 translocates to the nucleus, where it induces the transcription of numerous downstream target genes via their hypoxia-response elements 23. To date, the hypoxia response has been shown to control the GCC phenotype and to be associated with prognosis in gastric cancer patients 24,25. However, direct evidence is still needed to prove GCSPCs preferred reside in hypoxic PMSs, and the function of the hypoxic microenvironment and HIF regulation in GCSPC maintenance have not been fully elucidated.

In this study, we isolated GCSPCs from gastric cancer patients who suffered peritoneal dissemination using the side population approach. Using GCSPC-related protein markers and a pimonidazole label, we demonstrated that tumor stem cells were highly enriched in hypoxic regions of PMSs by a time-dependent GCPD mouse model. Moreover, we showed that hypoxia was involved in the regulation of GCSPC stemness through HIF-1α. These findings were further confirmed by the observation that GCPD was alleviated when the hypoxic microenvironment was destroyed in a mouse model. In summary, we identified the hypoxic niche as a critical regulator in GCSPC maintenance and provided new insights that may lead to successful targeting of GCSPCs in clinical treatment of gastric cancer.

## Materials and Methods

### Tissue Samples

Tumor specimens were obtained from 175 patients with gastric cancer who underwent surgery at the Department of Surgical Oncology, First Affiliated Hospital of China Medical University from 2003 to 2005. All patients underwent gastrectomy, and their clinical and pathological data were available. All surgical specimens were examined by experienced pathologists, and the distal resected margin was tumor-free. The study protocol was reviewed and approved by the Ethics Committee of China Medical University and all patients provided written informed consent to participate in the study.

### Immunohistochemical Staining of Tissue Samples

For immunohistochemical staining, 4-µm histological sections were deparaffinized with xylene and rehydrated through a graded series of alcohol. The sections were then boiled for 10 minutes in 0.01 M citrate buffer, and endogenous peroxidase activity was blocked by incubation in 0.3% H_2_O_2_ in methanol for 30 minutes. The sections were incubated overnight at 4°C with mouse monoclonal anti-HIF-1α antibodies (diluted 1:200; Abcam, Hong Kong, China, http://www.abcam.cn/hif-1-alpha-antibody-h1alpha67-chip-grade-ab1.html), mouse monoclonal anti-Nestin antibodies (diluted 1:500; Chemicon, http://www.emdmillipore.com/CN/zh/product/Anti-Nestin-Antibody%2C-clone-10C2%2C-Biotin-Conjugate,MM_NF-MAB5326B), rabbit polyclonal anti-Oct4 antibodies (diluted 1:400; Abcam, http://www.abcam.cn/oct4-antibody-ab18976.html), or rat polyclonal anti-CD68 antibodies (diluted 1:200; Abcam, http://www.abcam.cn/cd68-antibody-ab125212.html). The sections were exposed to biotin-labeled secondary antibodies for 1 hour and then developed with DAB-H_2_O_2_. Staining was scored on the following scale: 0, no staining; 1+, minimal staining; 2+, moderate to strong staining in at least 20% of cells; 3+, strong staining in at least 50% of cells. Cases with 0 or 1+ staining were classified as negative, and cases with 2+ or 3+ staining were classified as positive.

### Primary GCC Culture and Hypoxia

Primary GCC culture was described previously 26. Primary GCC lines were cultured from seven patients who suffered GCPD; patients provided written informed consent for participation in the study. For normoxic GCC culture, cells were cultured in a 5% CO_2_ humidified atmosphere at 37°C in Dulbecco's modified Eagle's medium (DMEM)/F-12 (Invitrogen, Carlsbad, CA, http://www.lifetechnologies.com/order/catalog/product/11330032?ICID=search-product) supplemented with 10% fetal bovine serum (FBS; Invitrogen, https://www.lifetechnologies.com/cn/zh/home/life-science/cell-culture/mammalian-cell-culture/fbs.html?icid=fr-fbs-main%20). For hypoxic GCC culture, cells were cultured in DMEM/F-12 supplemented with 1% FBS, placed in a sealed incubator chamber (Stem Cell, Canada) flushed with 1% O_2_, 5% CO_2_, and 94% N_2_ and were incubated at 37°C for the indicated times. GCC line 1 (GC1) and GCC line 2 (GC2) showed the best responses to hypoxic conditions, as measured by upregulation of HIF-1α in Western blotting; therefore, GC1 and GC2 were selected for further studies.

### Lentivirus Production and Infection of GCCs

The shRNA sequences directed against HIF-1α (shHIF) were shHIF1: AACCGGTTGAATCTTCAGATAT, shHIF2: AACCTCAGTGTGGGTATAAGA, and shHIF3: AAGACCGTATGGAAGACATTA, and the scramble sequence was CCGGGGGTCTGTATAGGTGGAGAC. Lentivirus construction was carried out by Genepharma (Shanghai, China). Primary GCCs were infected, and stable transfectants were selected in puromycin for 7 days. After this period, cells were expanded and exposed to normoxic or hypoxic conditions for 24 hours to test HIF-1α downregulation. The shHIF1 construct was the most efficient for downregulation of HIF-1α expression in GCCs based on Western blot analysis.

### Western Blotting

GCCs were washed in ice-cold phosphate-buffered saline (PBS) twice and collected using a cell scraper. Fifty microliters of RIPA buffer, supplemented with 1 mM PMSF, 1 µg/ml leupeptin, 1 mM β-glycerophosphate, 2.5 mM sodium pyrophosphate, and 1 mM Na_3_VO_4_, was added, and cells were incubated on ice for 20 minutes, followed by centrifugation for 20 minutes at 12,000*g* at 4°C. Next, 50 µg protein from each sample was resolved on 10% sodium dodecyl sulfate polyacrylamide gels and transferred to polyvinylidene fluoride membranes. Membranes were then blocked in Tris-buffered saline and Tween 20 (TBST) solution containing 4% skim milk for 2 hours at room temperature. Membranes were then incubated with mouse monoclonal anti-HIF-1α (diluted 1:5,000; Abcam), mouse monoclonal anti-E-cadherin (diluted 1:3,000; BD, San Jose, CA, http://www.abcam.cn/alpha-smooth-muscle-actin-antibody-ab5694.html), or rabbit polyclonal anti-α-smooth muscle actin (SMA; diluted 1:1,000; Abcam) antibodies in TBST solution overnight at 4°C. After washing three times in TBST, membranes were further incubated in horseradish peroxidase-conjugated secondary antibodies (diluted 1:5,000; Santa Cruz, http://www.scbt.com/datasheet-2005-goat-anti-mouse-igg-hrp.html) in TBST for 2 hours at room temperature. Membranes were incubated with ECL solution (Pierce) for 1 minute, and protein bands were visualized using the ECL chemiluminescence method.

### Boyden Chamber Migration Assay

Boyden chambers (BD) with 8-µm pore size polystyrene filter inserts for 24-well plates were used according to the manufacturer's instructions. Briefly, 2 × 10^4^ GCCs were seeded into the upper compartment of each chamber in 300 µl DMEM with 10% FBS. The chambers were placed into wells containing 750 µl of complete medium. The migration chambers were incubated for 24 hours in normoxic or hypoxic conditions at 37°C. Following incubation, the inserts were fixed and stained, and the number of migrating cells was counted as described. Two independent experiments were performed in duplicate. Images were collected and quantified using Image-Pro Discovery software (80i, Nikon, Japan).

### Cell Surface Staining Analysis

Surface staining of GCSPC-related proteins CD44 and lgr5 was analyzed by fluorescence-assisted cell sorting (FACS). Briefly, GCCs were harvested and fixed for 30 minutes. Cells were then washed twice with ice-cold PBS supplement with 1% bovine serum albumin. Incubation was carried out with rat monoclonal anti-CD44 (diluted 1:400; Abcam, http://www.abcam.cn/cd44-antibody-im7-fitc-ab19622.html) or rabbit polyclonal anti-lgr5 (diluted 1:200; Abcam, http://www.abcam.cn/gpcr-gpr49-antibody-ab75732.html) antibodies for 30 minutes at 4°C in the dark. GCCs were then incubated with FITC-conjugated donkey anti-rat (http://www.scbt.com/datasheet-2099-donkey-anti-mouse-igg-fitc.html) or FITC-conjugated donkey anti-rabbit secondary antibodies (both diluted 1:500; Santa Cruz, http://www.scbt.com/datasheet-2090-donkey-anti-rabbit-igg-fitc.html) for 30 minutes 4°C in the dark. Cells surface staining was determined by flow cytometry (FACS Caliber, BD).

### Side Population Analysis and Sorting

Sorting the side population of GCCs was described previously 27. Briefly, GCCs were harvested and resuspended at 1 × 10^6^ cells per milliliter in prewarmed 37°C DMEM/F-12 with 1% FBS. The cells were then labeled with Hoechst 33342 (Sigma-Aldrich, Saint Louis, MO) at a concentration of 5 µg/ml. The labeled cells were incubated in the dark for 75 minutes in a 37°C water bath with intermittent mixing, either alone or with 75 µmol/l verapamil (Sigma-Aldrich). The cells were resuspended in ice-cold PBS containing 1% FBS after staining and were maintained at 4°C until flow cytometry analysis. Stained cells were analyzed using a flow cytometer (FACS Aria II, BD).

### Clonogenic Assays

Cells were plated at 100 cells/well in six-well culture plates for 14 days with DMEM/F-12 supplemented with 10% FBS, and colonies were fixed with 4% paraformaldehyde and stained with 1% crystal violet. Images were collected using a digital microscope (80i, Nikon). The number of colonies formed (>2 mm diameter) was counted manually by three independent researchers.

### Tumorsphere Culture

GCSPC sphere medium was described previously 28.The medium consisted of serum-free DMEM/F-12 (Invitrogen), 1× B27 supplement (Invitrogen, https://www.lifetechnologies.com/cn/zh/home/brands/gibco/gibco-b-27-supplement.html?icid=fr-b27-main%20), 1× N2 supplement (Invitrogen, https://www.lifetechnologies.com/order/catalog/product/17502048?ICID=search-product), 50 ng/ml epidermal growth factor (Peprotech, http://www.lookchem.com/NewSell/memberproductdetail.aspx?spid=61216#.VA8sfPmSx3c), 100 ng/ml basic fibroblast growth factor (Peprotech, http://www.lookchem.com/NewSell/memberproductdetail.aspx?spid=10926523#.VA8spfmSx3c), 10 nM gastrin (Sigma-Aldrich, http://www.sigmaaldrich.com/catalog/product/sigma/scp0152?lang=zh&region=CN), and 100 ng/ml noggin (Peprotech). For primary generation tumorsphere culture, cells were diluted in GCSPC sphere medium (5,000 cells per milliliter) and plated at 500 µl per well in ultra-low attachment 24-well plates (Corning) in normal or hypoxic conditions. Cells were fed 50 µl GCSPC sphere medium every other day for 7 days. After 7 days, spheres were measured, and those measuring greater than 100 µm were counted as tumor sphere-forming units. Primary tumorspheres were collected after filtering through a 100-µm strainer (BD) and were then trypsinized with 0.5% trypsin-EDTA and resuspended at 5,000 cells per milliliter. Secondary and tertiary generations of tumorspheres were cultured in the same conditions as the primary generation. The data calculated for the number of the tumor spheres were the average of three independent experiments.

### Tumorsphere Immunofluorescence Staining and Observation by Confocal Microscopy

Spheroids were fixed with 4% paraformaldehyde for 24 hours, dehydrated through a graded series of sucrose, embedded in paraffin, and sectioned. Four-micron-thick sections were blocked with 10% goat serum for 30 minutes at room temperature then incubated with mouse monoclonal anti-HIF-1α (diluted 1:500; Abcam), mouse monoclonal anti-Nestin (diluted 1:200; Millipore, Bedford, MA), rabbit polyclonal anti-Oct4 (diluted 1:200; Abcam), mouse monoclonal anti-mucin5ac (diluted 1:100; Santa Cruz), or mouse monoclonal anti-mucin 6 (diluted 1:100; Santa Cruz, http://www.scbt.com/datasheet-71623-mucin-6-0-n-459-antibody.html) antibodies for 1 hour. After washing with PBS three times, sections were incubated with corresponding secondary Alexaflour 488 or 594 secondary antibodies (all diluted 1:200; Invitrogen, https://www.lifetechnologies.com/order/catalog/product/A14808?ICID=search-product). Nuclei were stained with DAPI (Sigma-Aldrich). Fluorescence staining was visualized using an immunofluorescence microscope (FV1000, Olympus, Japan). In all experiments, at least 10 spheroids per group were analyzed.

### Peritoneal Dissemination Model and Establishment of the Peritoneum Hypoxic Region

Eight- to ten-week-old male BALB/c nude mice (weighing 18–20 g each) were obtained from Vital River (Beijing, China). All animal protocols received prior approval from the China Medical University Animal Ethics Committee, and all experiments were performed in accordance with relevant guidelines and regulations.

To investigate the peritoneal hypoxic region and GCSPC distribution, a group of 15 mice were intraperitoneally (i.p.) injected with 1 × 10^4^ GC1_sp_s to establish a time-dependent GCPD model. Peritoneal tumor-bearing mice were sacrificed on days 7, 14, and 21 (five mice at each time point). The peritoneal hypoxic region was assessed by pimonidazole incorporation, as described previously 29. Briefly, 60 mg/kg pimonidazole hydrochloride (Chemicon) was injected intravenously (i.v.) into tumor-bearing mice. Two hours after injection, mice were sacrificed by cervical dislocation, and the peritoneal greater omentum was fixed and sectioned. The peritoneal hypoxic region was assayed by immunofluorescence confocal staining with a rabbit polyclonal anti-pimonidazole antibody (Chemicon).

### Immunofluorescence Staining and Confocal Microscopy of Tissue Samples

Four-micron-thick tissue samples were deparaffinized with xylene and rehydrated through a graded series of alcohol. The sections were then boiled for 10 minutes in 0.01 M citrate buffer, and endogenous peroxidase activity was blocked by incubation in 0.3% H_2_O_2_ in methanol for 30 minutes. Nonspecific binding was blocked by incubating slides with normal goat serum for 30 minutes at room temperature. Sections were incubated with rabbit polyclonal anti-Epcam (diluted 1:200; Abcam, http://www.abcam.cn/epcam-antibody-ab71916.html), rat polyclonal anti-CD68 (diluted 1:200; Abcam, http://www.abcam.cn/cd68-antibody-ab125212.html), rabbit polyclonal anti-Nestin (diluted 1:200; Abcam), rabbit polyclonal anti-Oct4 (diluted 1:200; Abcam), or rabbit polyclonal anti-pimonidazole (diluted 1:50; Chemicon) antibodies overnight at 4°C. After washing with PBS three times, sections were incubated with corresponding secondary Alexaflour 488 or 594 secondary antibodies (all diluted 1:200; Invitrogen). Nuclei were stained with DAPI. The stained sections were mounted and viewed under an immunofluorescence microscope (FV1000, Olympus, Japan).

### PMS Macrophage Depletion and Tumor Xenografts

For peritoneal depletion of macrophages, mice were treated with i.p. injections of clodronate liposomes (Encapsula Nano Sciences) at 25 mg/kg every 3 days, as described previously 30. After 9 days (three injections), mice were sacrificed by cervical dislocation. The greater omentum was excised, fixed, and sectioned. PMS macrophage quantity and PMS area were assayed by immunohistochemistry. Major axis (*L*) and minor axis (*S*) of PMS were measured, the area of milky spots was calculated by the formula: area (mm^2^) = *π* × *L* × *S*. For tumor xenograft, 1 × 10^4^ GC1_sp_s or GC1HIF-1α^Δ^_sp_s were i.p. injected into normal (np) or PMS macrophage-depleted (dp) mice. Ten mice were included in each of the four groups. Mice were sacrificed by cervical dislocation 30 days after inoculation. Ten minutes before sacrifice, mice were anesthetized via inhalation of isoflurane to reduce the suffering. GCPD status was then estimated by measuring the volume of ascites and counting the number of metastatic nodules in the peritoneum.

### Statistics

All statistical analyses were performed using SPSS 16.0 software. Overall survival rates were determined using Kaplan-Meier curves, with the event being defined as death related to cancer. The log-rank test was used to identify differences between the survival curves of different patient groups. In univariate analysis, continuous data obtained from two groups were analyzed by two-tailed Student's *t* tests, continuous data from three or more separate groups were analyzed by analysis of variance, and categorical data were analyzed by two-tailed *χ*^2^ tests. All data were expressed as means ± SDs. Statistical significance was set at an alpha value of *p* < .05.

## Results

### Hypoxia-Inducible Proteins Were Overexpressed in Gastric Cancer and Correlated with Stem Cell-Related Protein Expression and GCPD

**Figure 1:**
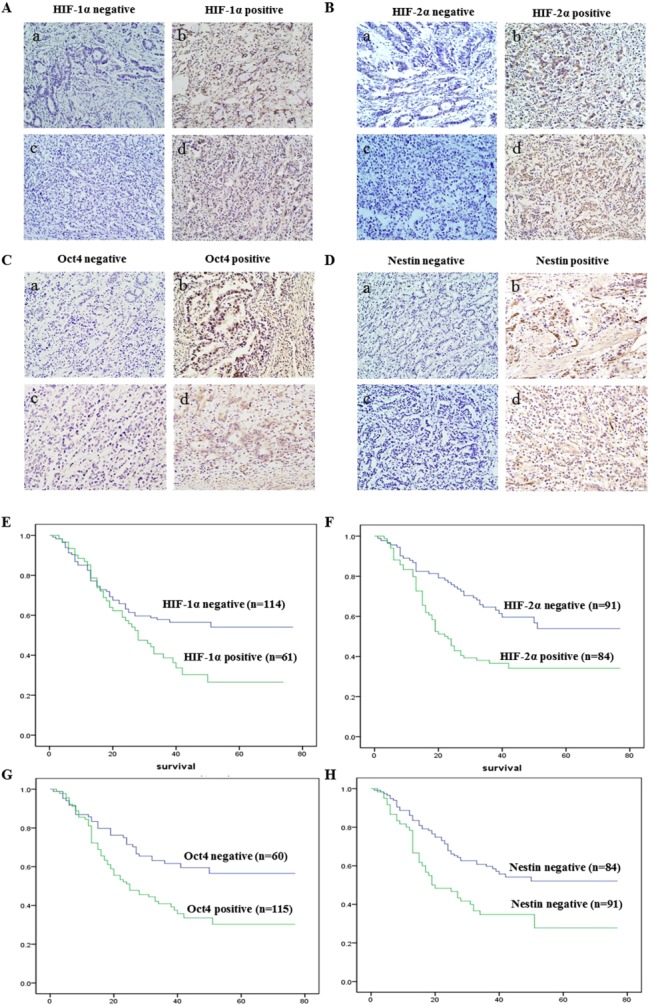
Hypoxia-inducible proteins were overexpressed in gastric cancer and correlated with stem cell-related protein expression and gastric cancer peritoneal dissemination. **(A):** HIF-1α expression was analyzed by immunohistochemical in 175 patients with gastric cancer (a, b were the samples from differentiated gastric cancer and c, d were the samples from undifferentiated gastric cancer, magnification ×600). **(B):** HIF-2α expression was analyzed by immunohistochemical in 175 patients with gastric cancer (a, b were the samples from differentiated gastric cancer and c, d were the samples from undifferentiated gastric cancer, magnification ×600). **(C):** Stem cell-related protein Oct4 expression was analyzed by immunohistochemical in 175 patients with gastric cancer (a, b were the samples from differentiated gastric cancer and c, d were the samples from undifferentiated gastric cancer, magnification ×600). **(D):** Stem cell-related protein Nestin expression was analyzed by immunohistochemical in 175 patients with gastric cancer (a, b were the samples from differentiated gastric cancer and c, d were the samples from undifferentiated gastric cancer, magnification ×600). **(E):** Overall survival rates from 175 gastric cancer patients were determined according to HIF-1α expression by Kaplan-Meier curves, with the event being defined as death related to cancer (114 patients showed negative expression, 61 patients showed positive expression). The log-rank test was used to identify differences between the survival curves of different patient groups (*p* = .003). **(F):** Overall survival rates from 175 gastric cancer patients were determined according to HIF-2α expression by Kaplan-Meier curves, with the event being defined as death related to cancer (91 patients showed negative expression, 84 patients showed positive expression). The log-rank test was used to identify differences between the survival curves of different patient groups (*p* = .002). **(G):** Overall survival rates from 175 gastric cancer patients were determined according to Oct4 expression by Kaplan-Meier curves, with the event being defined as death related to cancer (60 patients showed negative expression, 115 patients showed positive expression). The log-rank test was used to identify differences between the survival curves of different patient groups (*p* = .025). **(H):** Overall survival rates from 175 gastric cancer patients were determined according to Nestin expression by Kaplan-Meier curves, with the event being defined as death related to cancer (84 patients showed negative expression, 91 patients showed positive expression). The log-rank test was used to identify differences between the survival curves of different patient groups (*p* = .006). Abbreviation: HIF-1α, hypoxia-inducible factor-1α.

In order to investigate the correlation between the expression of hypoxia-inducible proteins and the stem cell-related proteins Oct4 and Nestin, we measured HIF-1α, HIF-2α, Oct4, and Nestin expression in 175 gastric cancer tissue samples by immunohistochemical staining. Figure 1A–1D illustrates HIF-1α, HIF-2α, Oct4, and Nestin expression in both differentiated and undifferentiated gastric cancer. Statistical analysis revealed that hypoxia-inducible proteins expression was associated with both Oct4 and Nestin expression as well as metastasis patterns in gastric cancer patients (Table1). Patients expressing high levels of HIF-1α protein were also found to have elevated Oct4 and Nestin expression (*p* = .006 and .046, respectively). Importantly, the expression of HIF-1α protein was significantly correlated with the development of GCPD (*p* = .018). Oppositively, patients expressing high levels of HIF-2α protein were found to have elevated Oct4 expression but no relation with Nestin expression (*p* < .001 and *p* = .175, respectively). Moreover, the expression of HIF-2α protein was shown to have no significantly relation with gastric cancer metastasis patterns (*p* = .824 for hematogenesis dissemination and *p* = .377 for GCPD, respectively). Calculation of the survival times of the 175 patients using the Kaplan-Meier method revealed that patients who harbored HIF-1α- or HIF-2α-positive tumors both had a shorter survival than patients who harbored HIF-1α- or HIF-2α-negative tumors (Fig. 1E, 1F, *p* = .003 and *p* = .017, respectively). Patients' survival times were also determined according to stem cell-related protein expression, as shown in Figure 1G, 1H, Oct4- and Nestin-positive expression were both negative prognostic factor in patients with gastric cancer (*p* = .025 and *p* = .006, respectively).

**Table 1:** Correlation between hypoxia-inducible factors expression and clinic pathological characteristic of gastric cancer patients

	HIF-1α expression			HIF-2α expression		
	Negative (*n* = 114)	Positive (*n* = 61)	*p* value	Negative (*n* = 91)	Positive (*n* = 84)	*p*-Value
OCT4 expression			.006^a^			<.001^a^
Negative	46	14		44	16	
Positive	68	47		47	68	
Nestin expression			.046^a^			.175
Negative	61	23		39	45	
Positive	53	38		52	39	
Hematogenesis dissemination			.524			.824
Negative	101	52		79	74	
Positive	13	9		12	10	
Peritoneal dissemination			.018^a^			.377
Negative	93	40		72	61	
Positive	21	21		19	23	
						

a , *p* < .05.

Abbreviation: HIF-1α, hypoxia-inducible factor-1α.

### Hypoxia Induced Epithelial-Mesenchymal Transition in GCCs and Enhanced the GCSPC Ratio in GCCs Through HIF-1α

**Figure 2:**
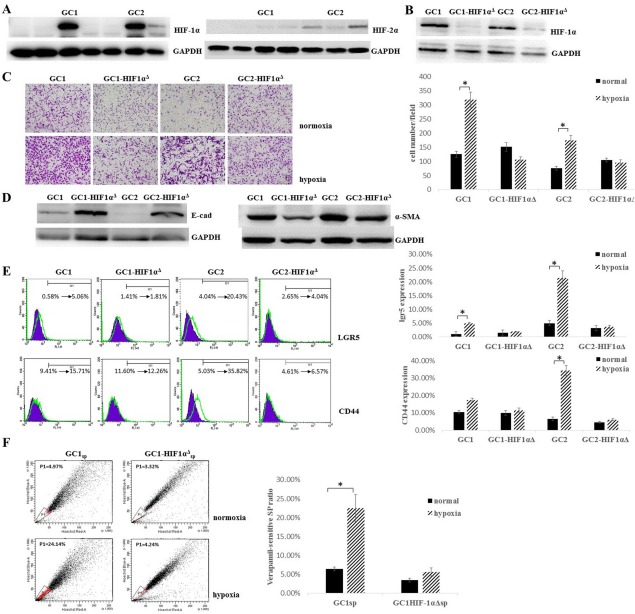
Hypoxia induced epithelial-mesenchymal transition in gastric cancer cells (GCCs) and enhanced the gastric cancer stem/progenitor cell ratio in GCCs through HIF-1α. **(A):** GCCs were primary cultured from seven patients who exhibited gastric cancer peritoneal dissemination, HIF-1α and HIF-2α expression were analyzed by Western blot after 48 hours hypoxic culture. GAPDH served as a protein loading control. **(B):** GC1 and GC2 were stably transfected with HIF-1α shRNA (GC1HIF-1α^Δ^ and GC2HIF-1α^Δ^) or scramble sequence (GC1 and GC2), HIF-1α expression was analyzed by Western blot after 48 hours hypoxic culture. GAPDH served as a protein loading control. **(C):** GC1, GC2, GC1HIF-1α^Δ^, and GC2HIF-1α^Δ^ were cultured under normoxic or hypoxic condition, migration ability was analyzed by Boyden chamber migration assay, cells were fixed with 4% paraformaldehyde and stained with 1% crystal violet (magnification ×200, cell number per field was expressed as mean ± SD, *n* = 4, *, *p* < .05). **(D):** Epithelial-mesenchymal transition-related protein E-cadherin and α-smooth muscle actin expression were analyzed by Western blot. GAPDH served as a protein loading control. **(E):** GC1, GC2, GC1HIF-1α^Δ^, and GC2HIF-1α^Δ^ were cultured under normoxic or hypoxic condition, surface staining of cancer stem cells related proteins CD44 and lgr5 was analyzed by fluorescence-assisted cell sorting (FACS, ratio of positive stained cell were expressed as mean ± SD, *n* = 4, *, *p* < .05, purple plots indicated GCCs cultured under normoxia and green plots indicated GCCs cultured under hypoxia). **(F):** Verapamil-sensitive SP ratio in GC1 and GC1HIF-1α^Δ^ was analyzed by FACS, cells were cultured under hypoxic or normoxic conditions for at least 7 days (ratio of SPs was expressed as mean ± SD, *n* = 3, *, *p* < .05). Abbreviation: HIF-1α, hypoxia-inducible factor-1α.

In order to investigate the role of the hypoxic microenvironment and HIF-1α during GCPD, we performed primary cultures of GCCs from seven patients who exhibited GCPD. As shown in Figure 2A, GC1 and GC2 cells showed better responses to hypoxia, as measured by upregulation of HIF-1α expression. However, as the HIF-2α expression was measured, GC1 and GC2 cells exhibited no HIF-2α expression, only two in seven primary cultured GCCs showed weak HIF-2α expression. Next, we stably transfected GC1 and GC2 cells with shHIF; no elevated HIF-1α expression was observed in either GC1HIF-1α^Δ^ or GC2HIF-1α^Δ^ following hypoxic stimulation, indicating that HIF-1α was a key regulator of the response to hypoxic stimuli in GC1 and GC2 cells (Fig. 2B). To investigate the roles of HIF-1α and hypoxia in metastatic ability, migration ability was assayed using Boyden chamber assays under both hypoxic and normoxic conditions. In all cases, GC1 and GC2 cells showed significantly higher migratory capacity in hypoxic conditions than in normoxic conditions (2.8- and 2.4-fold increases, respectively; both *p* < .05, Fig. 2C). In contrast, GC1HIF-1α^Δ^ and GC2HIF-1α^Δ^ showed no significant differences in migrated cell numbers under both hypoxic and normoxic conditions. Because of the observed increase in migration ability, E-cadherin and α-SMA expression were also assayed to investigate whether GC1 and GC2 cells undergo epithelial-mesenchymal transition (EMT) under hypoxic conditions. As shown in Figure 2D, GC1 and GC2 cells exhibited downregulation of the epithelial marker E-cadherin and upregulation of the mesenchymal marker α-SMA, which are considered specific features of the EMT, compared to those of GC1HIF-1α^Δ^ and GC2HIF-1α^Δ^ cells under hypoxic conditions. Because the EMT has been shown to endow tumor cells stem-like properties 31,32, we next assayed the surface expression of CD44 and lgr5, which have been shown to be specific markers of GCSPCs 33–35. As shown in Figure 2E, GC1 and GC2 cells showed elevated lgr5 and CD44 expression under hypoxic conditions compared with those under normoxic conditions (8.7- and 5.1-fold increases for lgr5, both *p* < .05; 1.7- and 7.1-fold increases for CD44, *p* = .24 and *p* < .05, respectively). Moreover, GC1HIF-1α^Δ^ and GC2HIF-1α^Δ^ cells showed no significant differences in lgr5 or CD44 expression under both normoxic and hypoxic conditions. The side population (SP) of GCCs has been shown to be rich in GCSPCs, which have self-renewal and pluripotent differentiation abilities, as previously described 27,36. Therefore, we measured the verapamil-sensitive SP ratio in GC1 (GC1_sp_), GC2 (GC2_sp_), GC1HIF-1α^Δ^ (GC1HIF-1α^Δ^_sp_), and GC2HIF-1α^Δ^ (GC2HIF-1α^Δ^_sp_) which were cultured under hypoxic or normoxic conditions for at least 7 days. Under normoxic condition, GC1 and GC1HIF-1α^Δ^ cells were both rich in verapamil-sensitive SPs (6.4% and 3.5%, respectively), after 7 days hypoxic culture, SPs showed a 3.5- and 1.6-fold increases (for GC1, SP ratio was 6.4% ± 0.54% in normoxia and 22.46% ± 3.57% in hypoxia, *p* < .05, Fig. 2F). However, very few SP cells (both <0.5%, Supporting Information Fig. S1A) were isolated from GC2 or GC2HIF-1α^Δ^ cells, despite the observation that these cells exhibited high levels of CD44 and lgr5 expression.

### Hypoxia Enhanced GCSPC Self-Renew, But Reduced GCSPC Differentiation

**Figure 3:**
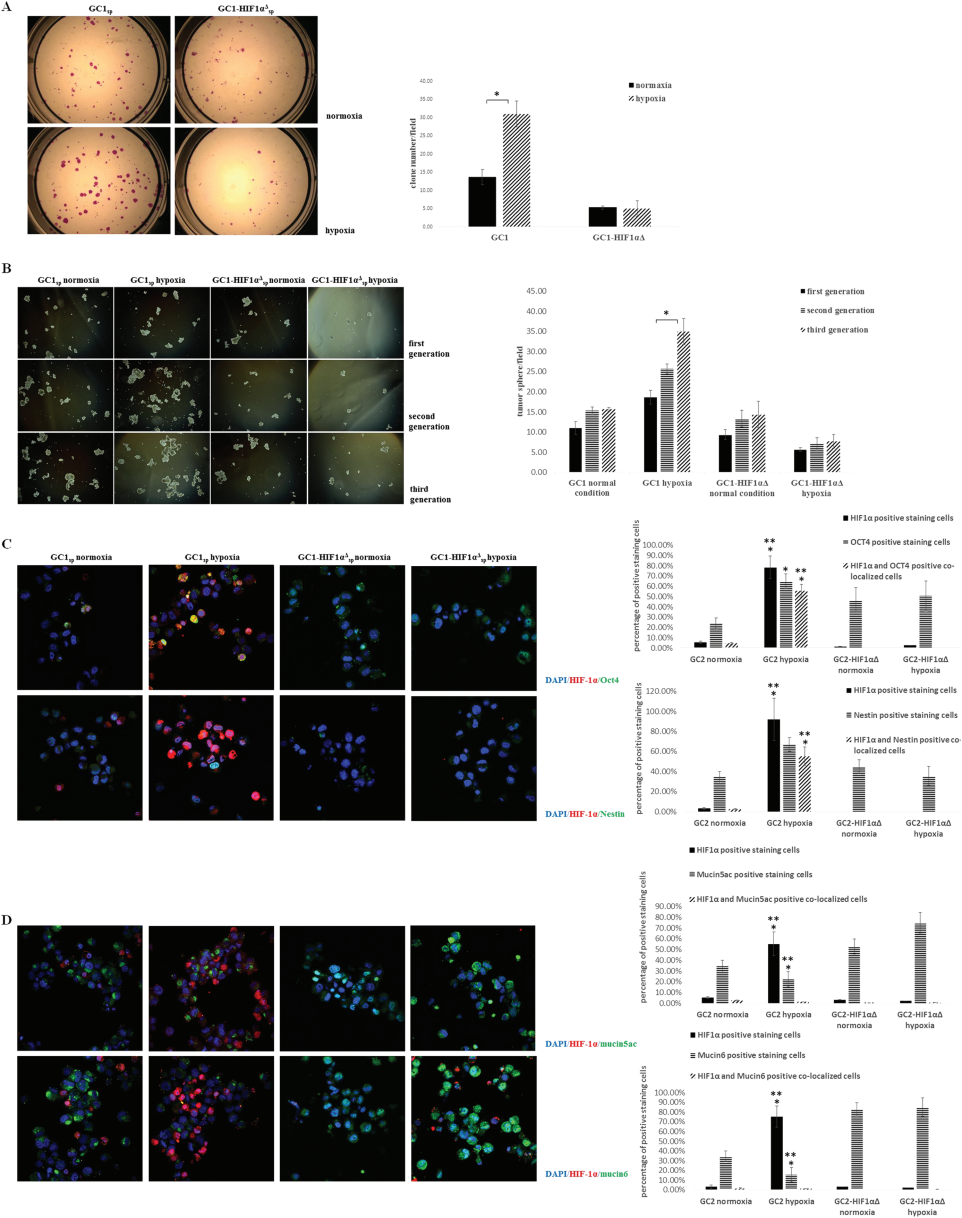
Hypoxia enhanced gastric cancer stem/progenitor cell (GCSPC) self-renew, but reduced GCSPC differentiation. **(A):** Side population of GC1 (GC1_sp_) and GC1HIF-1α (GC1HIF-1α^Δ^_sp_) were plated at 100 cells/well in six-well culture plates for 14 days with Dulbecco's modified Eagle's medium (DMEM)/F-12 supplemented with 10% fetal bovine serum and cultured under hypoxic or normoxic conditions, colonies were fixed and stained. The number of colonies formed (magnification ×40, >2 mm diameter) was counted manually by three independent researchers (number of colons were expressed as mean ± SD, *n* = 6, *, *p* < .05). **(B):** GC1_sp_ and GC1HIF-1α^Δ^_sp_ were diluted in GCSPC sphere medium (5,000 cells per milliliter) and plated at 500 µl per well in ultra-low attachment 24-well plates in normoxic or hypoxic conditions. The medium consisted of serum-free DMEM/F-12, 1× B27 supplement, 1× N2 supplement, 50 ng/ml epidermal growth factor, 100 ng/ml basic fibroblast growth factor, 10 nM gastrin, and 100 ng/ml noggin. Cells were fed 50 µl GCSPC sphere medium every other day for 7 days. Secondary and tertiary generations of tumor spheres were cultured in the same conditions as the primary generation (magnification ×60, numbers of tumorsphere were expressed as mean ± SD, *n* = 3, *, *p* < .05). **(C):** Spheroids of GC1_sp_ and GC1HIF-1α^Δ^_sp_ were fixed and sectioned, HIF-1α and stem-related protein Oct4 and Nestin expression were analyzed by immunofluorescence staining (DAPI showed blue staining, Oct4 and Nestin showed green staining, and HIF-1α showed red staining, magnification ×600, *, *p* < .05 compared with GC1_sp_ cultured under normoxia and **, *p* < .05 compared with GC1HIF-1α^Δ^_sp_ cultured under hypoxia). **(D):** Spheroids of GC1_sp_ and GC1HIF-1α^Δ^_sp_ were fixed and sectioned, HIF-1α and differentiation-related protein Mucin 5ac and Mucin 6 expression were analyzed by immunofluorescence staining (DAPI showed blue staining, Mucin 5ac and Mucin 6 showed green staining, and HIF-1α showed red staining, magnification ×600, *, *p* < .05 compared with GC1_sp_ cultured under normoxia and **, *p* < .05 compared with GC1HIF-1α^Δ^_sp_ cultured under hypoxia). Abbreviation: HIF-1α, hypoxia-inducible factor-1α.

We isolated the verapamil-sensitive SPs from GC1 and GC1HIF-1α^Δ^. Clone formation ability was assayed in GC1_sp_ and GC1HIF-1α^Δ^_sp_ cells under hypoxia and normoxia. All SP cells were shown to be heterogeneous 37,38, containing both stem/progenitor and mature/senescent cells. The variation tendency of stem/progenitor cells can be evaluated by colony formation assays because stem/progenitor cells are colony-forming cells. As shown in Figure 3A, GC1_sp_ cells formed more clones under hypoxic conditions when serum was added than those under normoxic conditions (31 ± 3.6 vs. 13.7 ± 2.1, *p* < .05). However, GC1HIF-1α^Δ^_sp_ cells showed no differences in clone numbers under both hypoxic and normoxic conditions (5.3 ± 0.5 vs. 5.1 ± 2.1). Serial passaging of tumor spheres was performed to assay the self-renewal ability of GCSPCs, and GC1_sp_ cells showed incremental tumor sphere formation from the first to the third generation under hypoxic conditions (Fig. 3B, *p* < .05). As expected, tumor sphere formation ability was stable in GC1_sp_ cells under normoxic conditions. Tumor sphere formation ability was also assayed in GC1HIF-1α^Δ^_sp_ cells, but no significant differences were found under both hypoxic and normoxic condition for all three generations (Fig. 3B). Clonogenic and tumorsphere formation assays were also carried out in GC2 cells. Consistent with GC1, hypoxia endowed GC2 an increased self-renewal ability through HIF-1α (Supporting Information Fig. S1B, S1C). To further confirm the presence of GCSPC in GC2, a gradient of 1 × 10^4^/10^5^/10^6^ GC2 or GC2HIF-1α^Δ^ was injected i.p. to the nude BALB/C mice. GC2 could generate metastatic nodules at a low GC2 gradient (Supporting Information Table S1). The expression levels of Oct4 and Nestin were assayed in single tumor spheres to verify the self-renewal ability of cells within the spheres using immunofluorescence staining and confocal imaging. Third-generation tumor spheres arising from GC1_sp_ cells cultured under hypoxic conditions showed greater numbers of Oct4- and Nestin-expressing cells compared with that of the other three groups. HIF-1α and Oct4 or Nestin were coexpressed in a subpopulation cells under hypoxic conditions (HIF-1α and Oct4 coexpressed in 55.66% ± 9.21% hypoxic cultured GC1 spheroid cells, HIF-1α and Nestin coexpressed in 45.41% ± 6.37% hypoxic cultured GC1 spheroid cells), these cells were considered GCSPCs (Fig. 3C). These results indicated that hypoxic environment endowed GCSPC stable and even growing self-renewal ability. Pluripotent differentiation potential is another feature of cancer stem cells 39. Therefore, we also evaluated the effects of hypoxic conditions on the expression of mucin5ac and mucin 6, specific markers of GCSPCs for foveolar and pyloric gland cell differentiation 40, in GCSPCs. As shown in Figure 3D, GC1_sp_ cells cultured under hypoxic conditions showed the lowest levels of mucin5ac and mucin6 expression (22.4% ± 7.14% for mucin5ac, 15.71% ± 6.28% for mucin6), indicating that these cells were immature/progenitor cells. Importantly, in the central region of the tumor sphere, no HIF-1α-expressing cells coexpressed mucin5ac or mucin6, suggesting that GCSPCs maintained an undifferentiated status.

### PMSs Served as a Hypoxic Niche During GCPD

**Figure 4:**
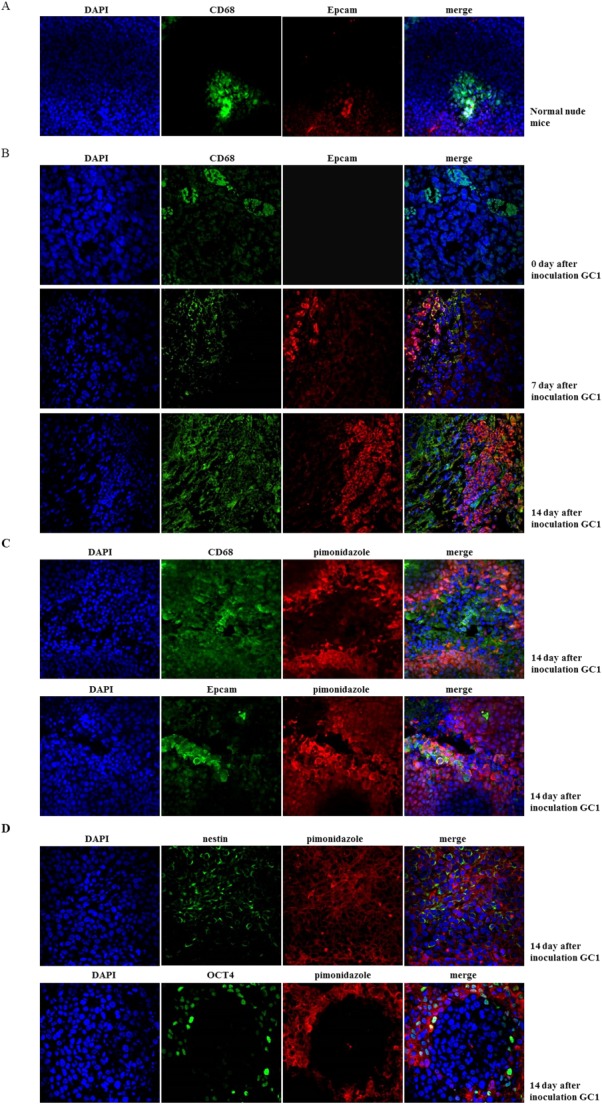
Peritoneal milky spots (PMSs) served as a hypoxic niche during gastric cancer peritoneal dissemination (GCPD). **(A):** Normal mouse peritoneum was fixed and sectioned. CD68 (marked PMS macrophage) and pimonidazole incorporation (marked hypoxia region) were analyzed by immunofluorescence staining (DAPI showed blue staining, CD68 showed green staining, and pimonidazole showed red staining, magnification ×400). **(B):** Fifteen male BALB/c nude mice (8–10 weeks old) were intraperitoneally (i.p.) injected with 1 × 10^4^ GC1_sp_s to establish a GCPD time-dependent model. Peritoneal tumor-bearing mice were sacrificed on days 7, 14, and 21 (five mice at each time point). Two hours before sacrifice, 60 mg/kg pimonidazole hydrochloride was injected intravenously (i.v.) into tumor-bearing mice to detect the hypoxic region. Mouse peritoneum was fixed and sectioned. CD68 and Epcam (marked GCSPC) expression were analyzed by immunofluorescence staining to mark macrophage and GCSPC, respectively (DAPI showed blue staining, CD68 showed green staining, and pimonidazole showed red staining, magnification ×400). **(C):** CD68, Epcam expression, and pimonidazole incorporation were analyzed by immunofluorescence staining (DAPI showed blue staining, CD68 and Epcam showed green staining, and pimonidazole showed red staining, magnification ×600). **(D):** Stem-related protein OCT4 and Nestin expression and pimonidazole incorporation were analyzed by immunofluorescence staining (DAPI showed blue staining, OCT4 and Nestin showed green staining, and pimonidazole showed red staining, magnification ×600).

Hypoxic PMSs have been suggested to be the origin of GCPD 14. We positioned the hypoxia region of PMS in untreated mice by confocal immunofluorescence staining. As shown in Figure 4A, hypoxic region located in the bottom of normal mice PMSs where newly formed blood and lymph capillaries were assembled 6. Next, we established a group of peritoneal tumor-bearing mice that received i.p. injections of 1 × 10^4^ GC1_sp_ cells in order to reveal the relationships between hypoxic regions and PMS distribution during GCPD. We discovered GCCs preferred anchorage-dependent growth within PMSs during GCPD. The areas of PMSs were enlarged after exposure to GCCs, and the margin of milky spots was blurred as GCC began clonal growth (Fig. 4B). Next, we investigated the positional relationships between hypoxic regions, PMS macrophages, and GCCs. We found that there was a band-like hypoxic region within the margins of PMSs. Most GCCs resided in the hypoxic region, while PMS macrophages were located in the center of the PMSs, in the vicinity of the hypoxic region (Fig. 4C). Regions of hypoxia are vital feature of the cancer stem cell niche (CSCN), and PMSs have also been speculated to represent a favorable microenvironment for GCSPC engraftment 11,12. Therefore, we wanted to investigate whether the localization of stem cell-related protein-expressing GCCs was in hypoxic regions. As shown in Figure 4D, the stem cell-related proteins OCT4 and Nestin were highly expressed in the hypoxic region, as compared to that in nonhypoxic regions.

### Block the Regulation of Hypoxia Microenvironment to GCSPC Alleviated GCPD in a Tumor Xenograft Model

**Figure 5:**
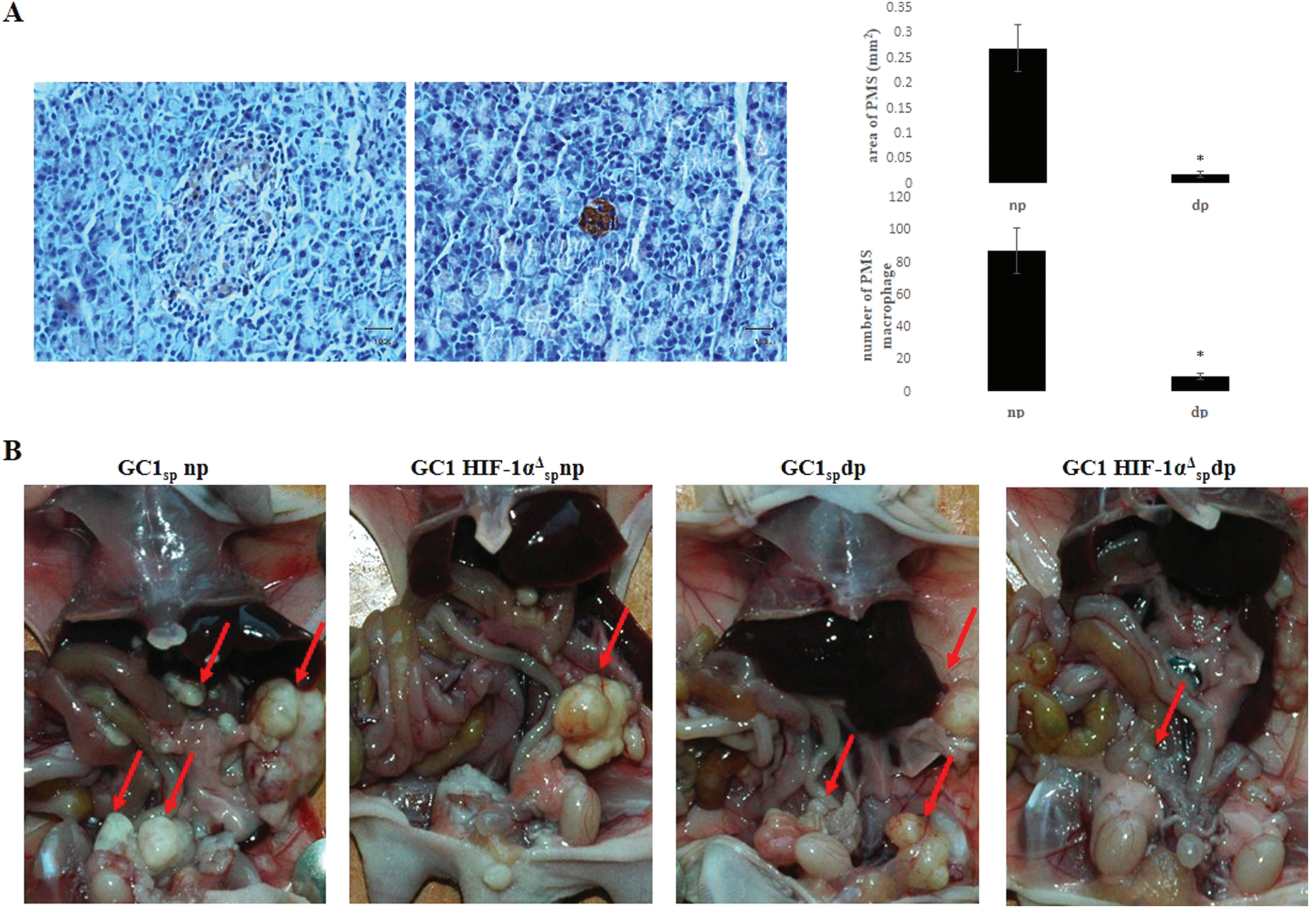
Block the regulation of hypoxia microenvironment to gastric cancer stem/progenitor cell alleviated gastric cancer peritoneal dissemination (GCPD) in a tumor xenograft model. **(A):** Mice were treated with i.p. injections of clodronate liposomes at 25 mg/kg every 3 days. After 9 days (three injections), mice were sacrificed by cervical dislocation. The greater omentum was excised, fixed, and sectioned. CD68 expression was analyzed by immunofluorescence staining to mark peritoneal milky spot (PMS) macrophage (major axis (*L*) and minor axis (*S*) of PMS were measured, the area of milky spots was calculated by the formula: area (mm^2^) = *π* × *L* × *S*, scale bar was 100 µM in length, *, *p* < .05). **(B):** 1 × 10^4^ GC1_sp_s or GC1HIF-1α^Δ^_sp_s were i.p. injected into normal (np) or PMS macrophage-depleted (dp) mice. Ten mice were included in each of the four groups. Mice were sacrificed by cervical dislocation 30 days after inoculation. GCPD status was then estimated by measuring the volume of ascites and counting the number of metastatic nodules in the peritoneum (red arrows showed metastatic nodules). Abbreviation: HIF-1α, hypoxia-inducible factor-1α.

PMSs are suitable for GCSPC engraftment, and GCSPCs resided in hypoxic PMSs expressed higher levels of stem cell-related proteins. We depleted PMS macrophages, which were considered to be the main cellular component of PMSs. IHC staining of CD68 showed significant reductions in both PMS macrophage numbers and the PMS area, which indicated a successful reduction and damage to PMSs (Fig. 5A). To exclude unintended effects of clodronate liposomes to GCCs, GCCs growth and invasion ability were examined when exposed to clodronate liposomes directly under both normoxia and hypoxia (Supporting Information Fig. S2). Normal dose (20 ng/ml) clodronate liposomes did not have significant effect to the growth and invasion ability of GCCs. To demonstrate whether damage PMS hypoxic microenvironment or blockage of HIF-1α transduction could efficiently reduce GCPD, 1 × 10^4^ GC1_sp_ or GC1HIF-1α^Δ^_sp_ cells were injected i.p. into np or dp mice. As shown in Figure 5B, the most severe GCPD occurred in the GC1_sp_ np group. In contrast, mice in the GC1_sp_ dp group exhibited reduced GCPD, confirming that PMSs served as a hypoxic niche and favored GCSPC self-renewal and outgrowth. In the GC1HIF-1α^Δ^_sp_ np group, GCPD was also alleviated compared that in the GC1_sp_ np group, which indicated that HIF-1α was a key regulator of the GCSPC phenotype in the hypoxic niche. As expected, the GC1HIF-1α^Δ^_sp_ dp group showed the fewest number of mice suffered with GCPD (Table2). All these results confirmed that PMSs served as a hypoxic niche and favored GCSPC engraftment and self-renewal, thus inducing GCPD through the regulation of HIF-1α.

**Table 2:** Gastric cancer peritoneal dissemination status of mice in each groups

		Normal PMS		PMS depletion		*p*-Value
		GC1	GC1 HIF-1α^Δ^	GC1	GC1 HIF-1α^Δ^	
Nodule formation	Yes	10	8	7	6	*p* < .05
	No	0	2	3	4	
Number of nodules		18 ± 3.3	12.7 ± 1.8	9.4 ± 2.7	8 ± 2.4	*p* < .05
Formation of bloody ascites	Yes	9	3	4	1	*p* < .05
	No	1	7	6	9	
Volume of bloody ascites (ml)		11 ± 2.1	5 ± 0.9	4 ± 1.3	3	*p* < .05

Abbreviations: HIF-1α, hypoxia-inducible factor-1α; PMS, peritoneal milky spot.

## Discussion

Gastric cancers are characterized by a hypoxic microenvironment, which correlates with tumor aggressiveness and poor outcomes. Overactivity of hypoxia-inducible proteins, sensor of low-oxygen conditions, is implicated in gastric cancer progression and metastasis 25,41,42. In our study, patients with HIF-1α- or HIF-2α-positive expression both had shorter survival times compared to patients with negative expression. However, increased incidence of GCPD was only discovered in HIF-1α-positive expression patients, indicating that HIF-1α is a predominant hypoxia sensor during GCPD process (Table1). The hypoxic microenvironment has been established its vital role in cancer stem cell regulation and maintenance 43,44. Thus, the expression of the stem cell-related proteins Oct4 and Nestin was also observed in gastric cancer patients. Patients with positive HIF-1α expression were also found to have elevated Oct4 and Nestin expression (Table1), indicating that hypoxia-regulated GCSPC maintenance may be involved in the GCPD process.

HIF-1α and HIF-2α are master regulator known to regulate various aspects of the cellular response to hypoxic stress 22–25. In this study, HIF-1α and HIF-2α expression were examined in GCCs primary cultured from seven GCPD patients. Strong HIF-1α expression was discovered in GC1 and GC2 cells; however, no remarkable HIF-2α was examined in all seven GCCs (Fig. 2A). Along with the results from gastric cancer patients tissue samples, we concluded that, HIF-1α and HIF-2α may both important regulator of GCCs behavior under hypoxia, HIF-1α seemed to be a predominant hypoxia regulator in milky spots hypoxic microenvironment during GCPD.

GC1 and GC2 showed enhanced expression of the GCSPC-related markers lgr5 and CD44 under a hypoxic microenvironment, indicating the presence of an elevated GCSPC ratio in the GCC population. To further investigate the effects of the hypoxic microenvironment on GC1 self-renewal and pluripotent differentiation ability, clonogenic and tumorsphere formation assays were carried out in vitro. We discovered that GC1 exhibited an enhanced self-renewal ability and was prone to maintain an undifferentiated status under hypoxic stress through HIF-1α (Fig. 3C, 3D). Even if no SPs were discovered in GC2, clonogenic and tumorsphere formation assays were also executed, because not all cancer stem cells must necessarily possess a SP phenotype. Consistent with GC1, hypoxia endowed GC2 an increased self-renewal ability through HIF-1α (Supporting Information Fig. S1B, S1C). In conclusion, our results demonstrated that the hypoxic microenvironment determined GCSPC fate and contributed to GCSPC survival and maintenance.

According to the “seed and soil” theory, metastases only occurs when tumor cells encounter a favorable microenvironment in which they can survive and proliferation 45. To trace GCSPCs during GCPD, we established a time-dependent mouse peritoneal dissemination model (Fig. 4B). We found that GCCs preferred anchorage-dependent growth within the hypoxic regions of PMSs in a time-dependent manner, which provided direct evidence that PMSs were an ideal niche whose hypoxic microenvironments favored GCPD. The anatomical environment includes cellular and acellular components that provide a highly specialized microenvironment for stem cell maintenance; this is defined as the CSCN 46. Elucidation of the characteristics of the CSCN and regulators of CSCN biology has been a subject of intense research. Initial reports have primarily focused on tumor vasculature, and a perivascular CSCN was observed in a glioma model 47. Recently, hypoxia, a common characteristic of solid malignant tumors, has been proposed as another feature of the CSCN 48,49. In our study, we demonstrated that GCSPCs were located in particular microenvironments within the PMS, which was consistent with the CSCN feature described above. Specifically, we showed that GCSPCs were located within the hypoxic margins of PMSs. Expression of the stem cell-related proteins Oct4 and Nestin was also higher in hypoxic regions than in nonhypoxic regions (Fig. 4C, 4D). To fully state PMSs are hypoxic, we also conducted a confocal immunofluorescence staining in normal mice to show the hypoxic region. As shown in Figure 4A, hypoxic region located in the bottom of normal mice PMSs. When the GCSPC entered into PMSs, hypoxic region was enlarged and turn to a band-like outline surround the PMSs (Fig. 4C). Together, these findings suggested that GCSPCs in the peritoneal cavity promoted self-growth by entering pre-existing hypoxic niches in PMSs, which could support the maintenance of GCSPCs and eventually cause GCPD.

To further confirm that the hypoxic microenvironment in PMSs was the key regulator responsible for GCSPC maintenance, we selectively destroyed PMSs by macrophage depletion or blocked the hypoxic response by knockdown of HIF-1α expression in GCSPCs. Mice in the GC1_sp_ dp group exhibited reduced GCPD, which confirmed that PMSs served as a hypoxic niche and favored GCSPC self-renewal and outgrowth. In the GC1HIF-1α^Δ^_sp_np group, GCPD was also reduced compared to that in the GC1_sp_ np group, indicating that HIF-1α was a key regulator of the cellular response to hypoxic stress. Consistent with this, very slight GCPD was observed in the GC1HIF-1α^Δ^_sp_ dp group.

## Conclusions

In summary, our results suggested that the hypoxic microenvironment in PMSs regulated GCSPC proliferation and maintenance through HIF-1α both in vitro and in vivo. These results provided new insights into the mechanisms of GCPD, which may lead to the development of novel therapies for gastric cancer in clinical practice.

## Supporting Information

See http://www.StemCells.com for supporting information available online.
