# Supplementary material for: Peritoneal Milky Spots Serve as a Hypoxic Niche and Favor Gastric Cancer Stem/Progenitor Cell Peritoneal Dissemination Through Hypoxia-Inducible Factor 1α
Source: Stem Cells. 2014 Nov 26;32(12):3062–74. doi: 10.1002/stem.1816 (PMC4282537; doi:10.1002/stem.1816)
Supplement: Supplementary file 1 [file stem0032-3062-SD1.doc]

**Suppliment material and method**

**MTT assay**

GC1 and GC1HIF-1α^Δ^ variability was assessed using an MTT [3-(4, 5-dimethylthiazol-2-yl)-2, 5-diphenyltrazoliumbromide] assay. Approximately 1×10^4^ cells/well GC1 or GC1HIF-1α^Δ^ cells were seeded into 96-well culture plates and cultured in DMEM with 10% FBS under hypoxia or normoxia. 20ng/ml clodronate liposomes was added for indicated times. Then, cells were incubated with 20 μL MTT (10 mg/ml) for 4 h at 37°C, and 200 μL DMSO was pipetted to solubilize the formazan product for 20 min at room temperature. The optical density (OD) was determined using a spectrophotometer (Bio-800, Bio-Rad, USA) at a wavelength of 570 nm. Approximately 1×10^4^ cells/well of GC1 or GC1HIF-1α^Δ^ cells were cultured with serum-free DMEM medium as a control group. Cell proliferation was calculated using the following equation:

Cell proliferation rate (%) = OD (Experimental group – Control group) / OD control group × 100%

**Boyden chamber migration assay**

Boyden chambers (BD) with 8-µm pore size polystyrene filter inserts for 24-well plates were used according to the manufacturer’s instructions. Briefly, the upper compartment was coated with 50 µL Matrigel (diluted 1:2; BD), then 5 × 10^4^ GC1 or GC1HIF-1α^Δ^ in 300 µL DMEM were seeded into the upper compartment of each chamber. The chambers were placed into wells containing 750 µL of complete medium. After adding 20ng/ml clodronate liposomes, the invasion chambers were incubated under indicated conditions for 24 h at 37°C under hypoxia or normoxia. Following incubation, the inserts were fixed and stained, and the number of migrating cells was counted. Experiments were performed in duplicate. Images were collected and quantified by using Image-Pro Discovery software (80i, Nikon).

**Suppliment Table 1 number of mice encountered peritoneal dissemination in different GC2 gradient**

|  |  | 1 × 10^6^ | | | 1 × 10^5^ | | | 1 × 10^4^ | | |
| --- | --- | --- | --- | --- | --- | --- | --- | --- | --- | --- |
|  |  | GC2 | GC2HIF-1α^Δ^ | P value | GC2 | GC2HIF-1α^Δ^ | P value | GC2 | GC2HIF-1α^Δ^ | P value |
| Peritoneal dissemination | yes | 5 | 4 | P < 0.05 | 4 | 2 | P < 0.05 | 1 | 0 | P < 0.05 |
|  | no | 0 | 1 |  | 1 | 3 |  | 4 | 5 |  |

**Suppliment Figure legend**

**Suppliment Figure 1 Hypoxia enhanced GC2 self-renew**

**A,** Verapamil-sensitive SP ratio in GC2 and GC2HIF-1α^Δ^ were analyzed by FACS, cells were cultured under hypoxic or normoxic conditions for at least 7days (ratio of SPs were expressed as mean ± SD, n=3, * *p<0.05*).

**B,** GC2 and GC2HIF-1α were plated at 1000 cells/well in 6-well culture plates for 14 days with DMEM/F12 supplemented with 10% FBS and cultured under hypoxic or normoxic conditions, colonies were fixed and stained. The number of colonies formed (> 2mm diameter) was counted manually by 3 independent researchers (magnification 40×, number of colons were expressed as mean ± SD, n=6, * *p<0.05*).

**C,** GC2 and GC2HIF-1α^Δ^ were diluted in GCSPC sphere medium (2 × 10^4^ cells/mL) and plated at 500 μL per well in ultra-low attachment 24 well plates in normoxic or hypoxic conditions. The medium consisted of serum-free DMEM/F12, 1× B27 supplement, 1× N2 supplement, 50 ng/mL epidermal growth factor (EGF), 100 ng/mL basic fibroblast growth factor (bFGF), 10 nM gastrin, and 100 ng/mL noggin. Cells were fed 50 μL GCSPC sphere medium every other day for 7 days. (magnification 60×, numbers of tumorsphere were expressed as mean ± SD, n=3, * *p<0.05*).

**Suppliment Figure 2 Clodronate liposomes did not influence the growth and invasion ability of GC1**

**A,** GC1 and GC1HIF-1α proliferation rate was analyzed by MTT assay at indicated time (data was expressed as proliferation rate, n=3).

**B,** GC1 and GC1HIF-1α invasion ability was analyzed by Boyden chamber invasion assay after 24h normoxic or hypoxic culture (data was expressed as folds of change, n=3).
