# Supplementary figures and images for: Peritoneal Milky Spots Serve as a Hypoxic Niche and Favor Gastric Cancer Stem/Progenitor Cell Peritoneal Dissemination Through Hypoxia-Inducible Factor 1α
Source: Stem Cells. 2014 Nov 26;32(12):3062–74. doi: 10.1002/stem.1816 (PMC4282537; doi:10.1002/stem.1816)

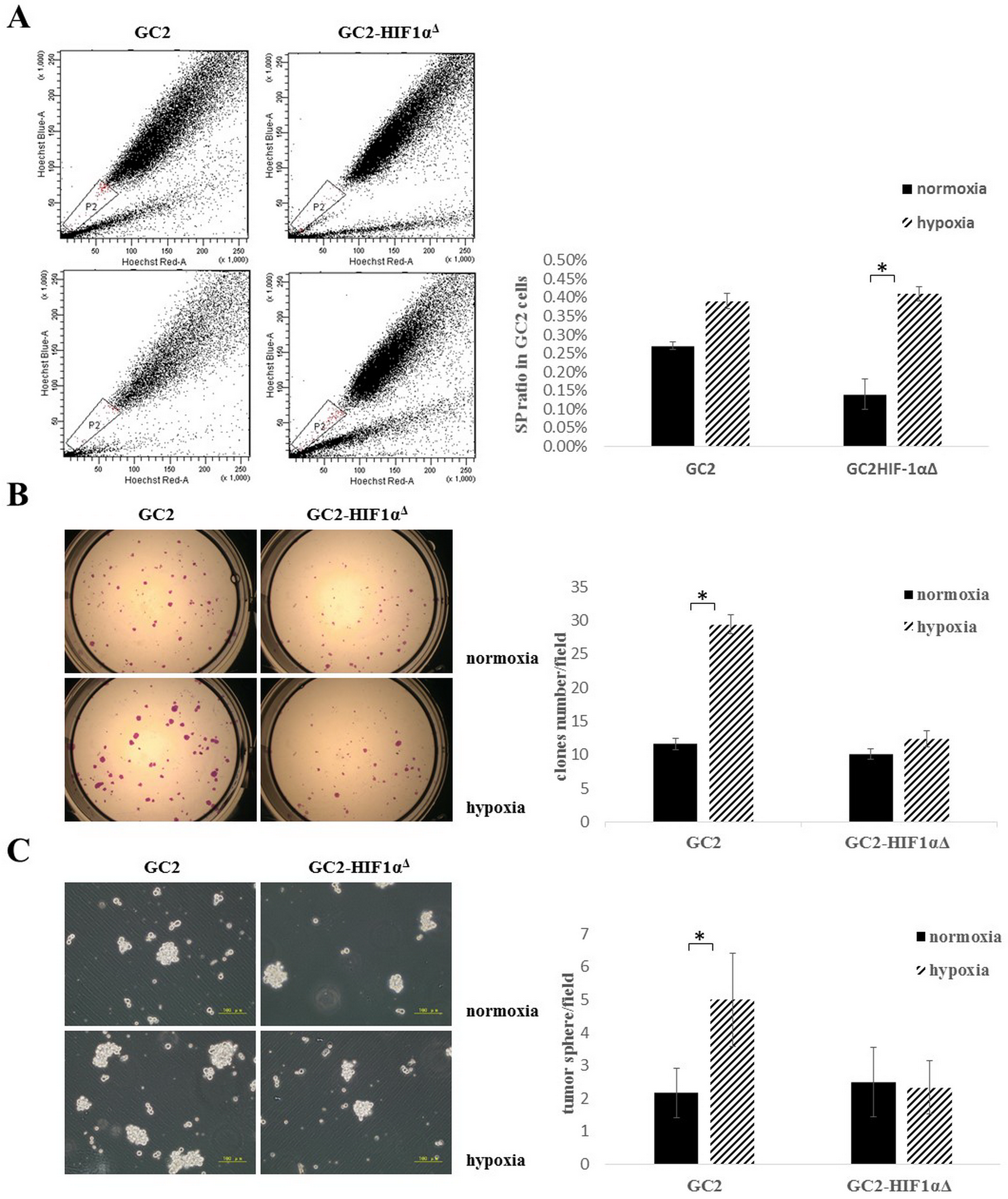

Supplement: Supplementary file 2 [file stem0032-3062-SD2.tif]

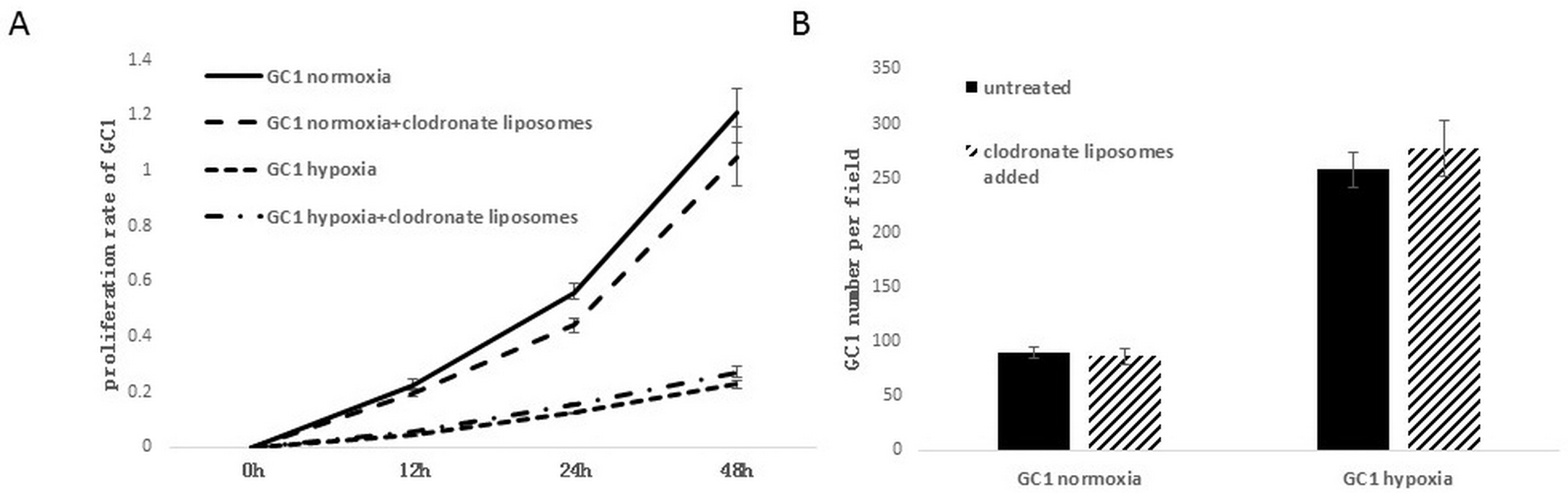

Supplement: Supplementary file 3 [file stem0032-3062-SD3.tif]
